# Environmental and Human Health Risks of 6PPD and 6PPDQ: Assessment and Implications

**DOI:** 10.3390/toxics13100873

**Published:** 2025-10-14

**Authors:** Sainan Zhang, Jiayue Tang, Zhiying Qiu, Xia Huo, Dongling Liu, Xiang Zeng

**Affiliations:** 1 School of Public Health, Zhejiang Chinese Medical University, 548 Binwen Road, Hangzhou 310053, China; 2 Laboratory of Environmental Medicine and Developmental Toxicology, Guangdong Key Laboratory of Environmental Pollution and Health, College of Environment and Climate, Jinan University, Guangzhou 511443, China; 3 School of Basic Medical Science, Zhejiang Chinese Medical University, 548 Binwen Road, Hangzhou 310053, China; 4 Zhejiang International Science and Technology Cooperation Base of Air Pollution and Health, 548 Binwen Road, Hangzhou 310053, China

**Keywords:** tire wear particles (TWPs), rubber additives, emerging contaminants, e-waste, microplastics, environmental persistence

## Abstract

This review aims to synthesize current knowledge on the environmental contaminants N-(1,3-dimethylbutyl)-N′-phenyl-p-phenylenediamine (6PPD) and its quinone derivative (6PPDQ) derived from tire wear particles (TWPs), focusing on their environmental distribution, transformation, human exposure pathways, toxicological effects, and health risks to ecological and human health. A comprehensive literature review was conducted, compiling and analyzing data from environmental monitoring studies, toxicological assessments on aquatic and mammalian models, and emerging human biomonitoring research. Key findings on concentrations, toxicological endpoints (e.g., LC50, oxidative stress, genotoxicity), and exposure pathways were evaluated. 6PPD and its transformation product 6PPDQ are ubiquitous environmental pollutants found in air, water, soil, sediment, and dust. 6PPDQ is notably highly toxic to aquatic organisms, with an acute LC50 of 790 ng/L for coho salmon. Human exposure to these compounds occurs through inhalation, ingestion, and dermal contact, and their presence has been confirmed in human matrices including blood, urine, and cerebrospinal fluid. Toxicological studies, primarily on model organisms, indicate that 6PPD and 6PPDQ can induce oxidative stress, cause DNA damage, and disrupt metabolic and neurological functions. Adverse outcomes such as intestinal toxicity, reproductive impairment, neurobehavioral changes, and potential carcinogenicity have been observed. However, direct evidence of their health impacts on humans remains limited. 6PPD and 6PPDQ pose significant and widespread ecological risks, with 6PPDQ representing a particularly potent aquatic toxicant. While human exposure is confirmed, the full scope of human health implications is not yet well understood. The review highlights the need for longitudinal environmental tracking, mechanistic studies, and refined exposure models to inform regulatory actions and mitigate risks. Addressing these challenges is essential to mitigate the ecological and health burdens posed by 6PPD and 6PPDQ. This study underscores the global societal importance of addressing 6PPD-related pollution—a pervasive and transboundary environmental challenge stemming from universal tire wear.

## 1. Introduction

The widespread application of rubber products in modern industrial and consumer applications, including tires, hoses, cables, footwear, and other various industrial/household items, has led to increasing concerns regarding their environmental persistence and associated health risks [1]. Rubber materials are susceptible to degradation thereby affecting their performance and service life under environmental stressors such as heat, UV radiation, and oxidative processes, resulting in cracking, hardening, and loss of elasticity [1,2]. The degradation of rubber originates from irreversible chemical changes in its molecular structure. Specifically, even low-level ozone exposure can trigger thermal-oxidative aging that causes either chain scission or cross-linking reactions of rubber double bonds [3]. Simultaneously, ultraviolet radiation exacerbates the deterioration by exciting photosensitive groups, thereby accelerating oxidation [4,5]. To mitigate these effects, antioxidants and antiozonants are routinely incorporated during manufacturing. Among these additives, N-(1,3-dimethylbutyl)-N′-phenyl-p-phenylenediamine (6PPD) and its ozonation by-product, 6PPD quinone (6PPDQ), are extensively utilized due to their high efficacy, particularly in tire production with typical values of 0.5–1.5 weight percent [6,7,8].

6PPD and 6PPDQ are characterized as gray-black solids with low water solubility but high solubility in organic solvents such as benzene and toluene [2]. Global production of 6PPD has increased substantially, with China alone manufacturing over 200,000 tons annually as of 2020 [9]. However, their extensive use has resulted in significant environmental release. Notably, the antiozonant activity of 6PPD derives from a direct chemical interaction of its aromatic ring with ozone, whereas this protective mechanism simultaneously leads to the formation of its ozone oxidation by-product of 6PPDQ [10]. This conversion yields a highly persistent, toxic compound, with severe ecological consequences for coho salmon, chum salmon, and zebrafish [11,12,13,14]. The molar yield of this conversion process is approximately 0.95% under typical environmental conditions [15]. In 2024, Zhu et al. demonstrated the extreme toxicity of 6PPDQ to silver salmon, with reported 24 h median lethal concentrations (LC50) as low as 790 ng/L, a toxicity level far exceeding that of several known pollutants [16]. Similarly, zebrafish embryos exposed to 6PPD exhibit significant developmental abnormalities, including reduced hatching success, impaired locomotor activity, spinal deformities, and pericardial edema [17,18]. Despite growing evidence of its ecological hazards such as acute toxicity, behavioral disruption, neurotoxicity, and immunotoxicity in model organisms, research on the human health implications of 6PPD and 6PPDQ exposure remains limited. Current understanding of their toxic mechanisms, long-term health risks, and potential mitigation strategies is still in its infancy [1,19].

In this study, we summarize existing knowledge on the environmental distribution, exposure pathways, and toxicological effects of 6PPD and 6PPDQ, with a particular focus on their impacts on aquatic organisms and potential human health risks. Furthermore, we evaluate current health risk assessment methodologies and propose potential protective measures to mitigate environmental and public health exposure. A comprehensive understanding of these compounds is critical for developing early-warning systems and preventive strategies to minimize their adverse effects. Consequently, this study highlights a critical global issue: tire wear pollution transcends national boundaries. Therefore, the insights presented here are vital for shaping proactive environmental and public health strategies on a global scale, calling for immediate and collaborative international attention.

## 2. Methodology

A comprehensive and systematic literature search was executed across multiple electronic databases, including Web of Science, PubMed, Science Direct, SpringerLink, Wiley Online Library, ACS Publications, and Google Scholar, for records published up to October 2025. Search strategies combined two conceptual categories: (1) 6PPD/6PPDQ: (“N-(1,3-dimethylbutyl)-N′-phenyl-p-phenylenediamine” OR “N-(1,3-dimethylbutyl)-N′-phenyl-1,4-benzenediamine” OR “N-(1,3-dimethylbutyl)-N′-phenyl-p-phenylenediamine-quinone” OR “6PPD” OR “6PPDQ” OR “6ppd” OR “6ppdq” OR “6PPD-Q” OR “6PPD-quinone” OR “6PPD quinone”); (2) Risk assessment: (“Risk assessment” OR “Risk evaluation” OR “Risk calculation” OR “Risk analysis” OR “Risk characterization” OR “Health risk assessment” OR “Health impact assessment”). This approach aimed to maximize retrieval sensitivity while minimizing exclusion bias. Two authors independently screened the identified studies to ensure selection consistency, with the search limited to English-language publications for analytical uniformity.

## 3. Environmental Distribution of 6PPD and 6PPDQ

**Figure 1:**
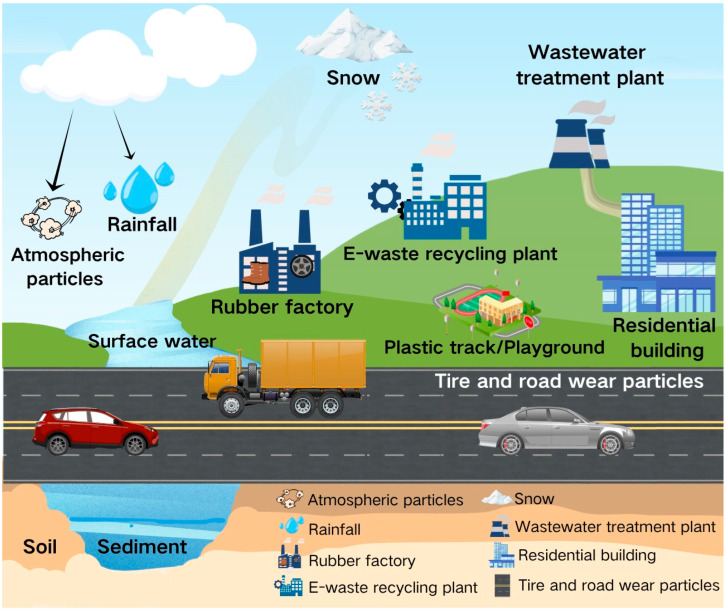
Sources and distribution of 6PPD and PPDQ in the environment.

The main sources of 6PPD and its transformation product 6PPDQ in the environment mainly include but are not limited to tire wear particles (TWPs), direct emissions from rubber products, recycled rubber materials, and electronic waste (e-waste) recycling. They are universally distributed in environmental media such as atmosphere, water, sediment, soil, and dust through different pathways (Table 1), directly or indirectly causing health hazards to living organisms (Figure 1).

### 3.1. Atmosphere

#### 3.1.1. Atmospheric Distribution and Dynamics of 6PPD and 6PPDQ

The atmospheric distributions of 6PPD and its derivative 6PPDQ are predominantly driven by their associated mainly with tire wear particles (TWPs). Continuous abrasion of rubber products, particularly vehicle tires, releases 6PPD into the environment, where it undergoes rapid oxidation upon reaction with atmospheric ozone to form 6PPDQ [20]. These compounds adsorb onto fine particulate matter (PM_2.5_) and respirable coarse particles (PM_10_), facilitating their long-range atmospheric transport [21]. Due to the low settling velocities and prolonged suspension of PM_2.5_ and PM_10_ in air, 6PPD and 6PPDQ exhibit extended atmospheric residence times, enhancing their potential for regional and even global distribution [22].

Empirical studies have confirmed the widespread presence of 6PPD and 6PPDQ in airborne particulates such as fine particles (PM_2.5_) and respirable particulate matter (PM_10_) across diverse urban environments with the wind and airflow. Zhang et al. conducted the first large-scale detection of 6PPDQ in PM_2.5_ samples from six major Chinese cities (Guangzhou, Hangzhou, Zhengzhou, Taiyuan, Shanghai, and Nanjing), reporting a detection frequency of 81% [23]. Wang et al. further corroborated these findings by identifying both 6PPD and 6PPDQ in PM_2.5_ collected in Hong Kong, underscoring their ubiquity in urban atmospheres [20].

The spatiotemporal variability of 6PPD and 6PPDQ in airborne particulates is modulated by multiple anthropogenic and environmental factors such as traffic flow, rubber product use patterns, climatic conditions, and geographic location. Elevated vehicular activity correlates with increased TWP emissions, directly augmenting atmospheric 6PPD/6PPDQ loads [24]. Higher concentrations are observed during winter months due to intensified heating demands and stagnant meteorological conditions that exacerbate particulate accumulation [25]. Wind patterns, precipitation, and urban topography govern particulate dispersion and deposition rates [22]. Non-tire sources (e.g., industrial rubber goods, artificial turf) contribute to background emissions, particularly in industrialized regions [6].

#### 3.1.2. Dust

Dust serves as a significant environmental sink for 6PPD and 6PPDQ with distinct distribution patterns between indoor and outdoor environments. Among outdoor dust matrices, road dust represents the dominant outdoor reservoir for these compounds, largely sourced from tire wear particles (TWPs) [20]. TWPs undergo environmental degradation to form PPD-derived quinones, with a substantial proportion accumulating on road surfaces and adjacent roadside dust. A fraction of this roadside dust can migrate into indoor environments through various pathways including particulate infiltration through building openings (doors, windows), deposition on outdoor floors and indoor surfaces (chair, table, bed), and transport or adhere via personal items (clothing, footwear) [26].

**Table 1:** The concentrations of 6PPD and 6PPDQ in air and dust in the worl32.

Environmental Media	Characteristics	6PPDQ	6PPD	References
Air	City	1.18 (0.54–13.8) pg/m^3^	1.78 (0.82–6.30) pg/m^3^	[27]
	City	0.85 (NQ–1.75) pg/m^3^	(NQ–<LOQ) pg/m^3^	[28]
	Guangzhou	1.7 (0.1–15) pg/m^3^	0.9 (0.3–10) pg/m^3^	[29]
	Hangzhou	6.7 (0.8–26) pg/m^3^	4.6 (0.1–6.0) pg/m^3^	
	Nangjing	2.3 (1.1–68) pg/m^3^	2.1 (0.4–75) pg/m^3^	
	Shanghai	5.9 (0.3–39) pg/m^3^	4.4 (0.5–135) pg/m^3^	
	Taiyuan	3.3 (1.1–84) pg/m^3^	6.9 (0.02–487) pg/m^3^	
	Zhengzhou	2.9 (0.3–32) pg/m^3^	8.4 (1.2–109) pg/m^3^	
	Guangzhou	1100 (3.04–2350) pg/m^3^	1820 (22.2–6050) pg/m^3^	[20]
	Roadside in Guangzhou	2810 (2.96–7250) pg/m^3^	4040 (2.23–9340) pg/m^3^	
	Taiyuan	744 (2.44–1780) pg/m^3^	81 (1.02–3190) pg/m^3^	
Dust	E-waste recycling workshops	375 (87.1–2850) ng/g	113 (13.8–1020) ng/g	[30]
	Playground	/	30.4 (<MQL–685) ng/g	[21]
	Indoor dust	/	16.4 (<MQL–180) ng/g	
	Air conditioner filters—male dormitories	4.76 ± 2.81 (1.95–13.4) ng/g	/	[23]
	Air conditioner filters—female dormitories	6.78 ± 2.98 (2.85–12.6) ng/g	/	
	Air conditioner filters—residential houses	11.4 ± 8.11 (0.62–31.7) ng/g	/	
	Settled dust—residential bedrooms	10.7 ± 7.58 (0.97–26.1) ng/g	/	
	Settled dust—buses	43.0 ± 12.9 (19.7–71.4) ng/g	/	
	Settled dust—shopping malls	23.5 ± 23.4 (3.92–106) ng/g	/	
	Vehicle dust	80.9 (17.9–146) ng/g	19.3 (5.0–41.9) ng/g	[31]
	House dust	<LOQ (<LOQ–0.4) ng/g	0.3 (<LOQ–6.1) ng/g	
	E-waste dust	/	15.4 (7.31–37.7) ng/g	[32]
	House dust (Canada)	/	0.083 (<MDL–6.65) ng/g	
	House dust (United States)	/	1.84 (<MDL–23.7) ng/g	
	indoor dust	9.5 (0.33–82) ng/g	10 (0.48–135) ng/g	[16]
	E-waste community indoor dust	3.2 ng/g	/	[33]
	E-waste kindergarten indoor dust	7.5 ng/g	/	
	Haojiang—house dust	1.4 ng/g	/	
	Haojiang—kindergarten dust	1.3 ng/g	/	

Previous studies have consistently detected both 6PPD and 6PPDQ across multiple dust compartments such as outdoor road dust and indoor dust. For example, KoLe et al. quantitatively detected 6PPDQ in road dust and indoor parking lot dust in Guangzhou [34]. Notably, 6PPDQ has also been identified in various nonoccupational settings, including vehicles, dormitories, residential dwellings, air-conditioned spaces, and shopping malls, indicating its widespread distribution [31,35]. Furthermore, environmental conditions such as elevated temperatures and ozone exposure can accelerate the release of these compounds into the surrounding environments [34].

Both 6PPD and 6PPDQ exhibit similar distribution trends across indoor and outdoor dust samples, regardless of regional differences. However, major road dust, particularly from high-traffic areas such as highways, remains the dominant reservoir for these compounds, as evidenced by significantly higher 6PPDQ concentrations in road dust compared to indoor samples [23]. Additionally, their concentrations and detection frequencies exhibit a declining gradient along the pathway of “main road dust > residential road dust > residential square dust > green belt topsoil” [19]. Nevertheless, this spatial trend is not entirely consistent across different regions, as the distribution of 6PPD and 6PPDQ in dust is also influenced by factors such as geographic location, rubber product prevalence, and atmospheric circulation [19].

For example, Liang et al. (2022) reported a median 6PPDQ concentration of 375 ng/g in dust from e-waste dismantling workshops, far exceeding typical outdoor dust levels [30]. Similarly, Huang et al. observed that the median concentrations of 6PPD and 6PPDQ in outdoor road dust (52.5 ng/g and 32.2 ng/g, respectively) substantially exceeded those in indoor living room dust (0.3 ng/g and <LOQ, respectively) [31]. However, higher levels of 6PPD and 6PPDQ in air were detected in parking lots and vehicle interiors than those in roadway dust, which was likely due to the accumulation of TWPs in confined, poorly ventilated spaces.

### 3.2. Water Environment

Atmospheric 6PPD and 6PPDQ can enter aquatic ecosystems through rainfall, surface runoff, and direct deposition into water bodies. The toxicity of 6PPD and 6PPDQ to aquatic organisms cannot be ignored (Table 2). The presence and distribution of 6PPD and 6PPDQ in aquatic environments have become a major focus of environmental research [36,37,38,39]. In addition, rainfall serves as a key transport pathway, facilitating the movement of these contaminants into surface waters, where they accumulate in rivers, lakes, and runoff systems [40].

Of particular concern, Tian et al. revealed through runoff modeling that predicted 6PPDQ concentrations in river and lake water across multiple monitored cities exceeded the newly revised LC50 threshold, which sounded an alarm for the ecological risk caused by 6PPDQ [2]. Furthermore, Zhou et al. found that TWPs exhibit density-dependent behavior in aquatic systems: denser particles settle into sediments, while lighter fractions are transported via runoff into rivers [50]. Consequently, 6PPD and 6PPDQ have been detected in various aquatic matrices, including river surface water, rainwater, and snowmelt [51]. However, compared to more commonly studied environmental samples (e.g., river water and rainfall), research on their presence in snowmelt remains limited [9,52].

During rainfall events, 6PPD and 6PPDQ are transported through urban drainage systems, ultimately entering municipal wastewater treatment plants (WWTPs). Research indicates these compounds persist throughout the treatment process, with detectable levels found in both effluent and biosolids (Table 3). For instance, Johannessen et al. (2021a) reported an average 6PPD concentration of 0.05 µg/L near WWTP outfalls—exceeding upstream and downstream levels that fell below detection limits [28]. Notably, Cao et al. (2022) confirmed the presence of both compounds in Hong Kong WWTPs across all treatment stages (influent, effluent, and biosolids), suggesting conventional wastewater treatment processes may be insufficient for complete removal [27]. This persistence raises dual concerns: environmental risks through downstream contamination of receiving waters; potential human health implications via biosolid-amended soils or water reuse (Table 4).

### 3.3. Soil Environment

Emerging research has documented the widespread presence of 6PPD and its transformation product 6PPDQ in terrestrial environments, with particularly high accumulation observed in soil matrices. These compounds demonstrate significant environmental partitioning behavior, with recent studies detecting them in riverine sediments from the Pearl River Delta and Pepper River watersheds [9]. Wastewater treatment processes appear to enhance this soil accumulation, as evidenced by the higher retention of 6PPDQ in sludge (20.0%) compared to aqueous phases (16.9%) in WWTP systems [9].

The distribution of 6PPD and 6PPDQ in soils exhibits substantial variability, with concentrations reaching up to 309 ng/g and 234 ng/g in roadside soils, respectively [27]. This spatial heterogeneity is likely driven by anthropogenic factors, particularly vehicular traffic and tire wear. Wagner et al. propose that tire-derived particles, generated through road friction, serve as a primary emission pathway for rubber additives such as 6PPD and 6PPDQ [70]. Supporting this hypothesis, Huang et al. observed higher levels of these compounds in agricultural topsoils adjacent to highways compared to residential green belts, reinforcing the role of automobile proximity in contamination patterns [31].

The pervasive presence of 6PPD and 6PPDQ in soils raises concerns regarding their ecotoxicological effects. They may pose direct hazards to soil-dwelling organisms, including earthworms, and can accumulate in plants via root uptake [71]. Furthermore, 6PPDQ exhibits differential toxicity toward soil microbiota, with fungi displaying greater sensitivity than bacteria. Intriguingly, Wu et al. (2024) suggest that 6PPDQ accumulation may alter soil carbon dynamics, particularly during winter, highlighting its potential to disrupt biogeochemical cycles [72]. Collectively, these findings underscore the emerging threat of 6PPD and 6PPDQ to soil ecosystem integrity and necessitate further research into their long-term ecological consequences (Table 5).

### 3.4. Special Exposure Scenarios of 6PPD and 6PPDQ Derived from E-Waste

Beyond typical daily exposure, e-waste dismantling activities represent a significant yet understudied source of 6PPD and 6PPDQ contamination. Research indicates that concentrations of these compounds are substantially higher in e-waste dismantling areas compared to non-dismantling areas. For instance, Zhang et al. reported that atmospheric levels of 6PPD and 6PPDQ were significantly higher in an e-waste recycling area (Guiyu) than the adjacent reference region (Haojiang) [33]. Moreover, Dai et al. also reported that the urinary levels of 6PPD and 6PPDQ in 98 children were greater in Guiyu than their peers in Haojiang [9]. This suggests that e-waste processing contributes to direct human intake, likely through inhalation or dust ingestion.

The extent of contamination varies depending on management practices and environmental conditions. Notably, dust and soil from modern e-waste dismantling parks exhibit lower 6PPD and 6PPDQ levels than traditional informal e-waste recycling sites, likely due to improved waste-handling protocols [74]. Additionally, seasonal fluctuations influence concentrations, with peak levels observed in winter, possibly due to reduced degradation under lower temperatures and UV exposure [74].

These findings highlight e-waste recycling as a critical yet overlooked exposure pathway for 6PPD and 6PPDQ, warranting further investigation into mitigation strategies and health risks for workers and nearby residents (Table 6).

## 4. Human Exposure Routes

**Figure 2:**
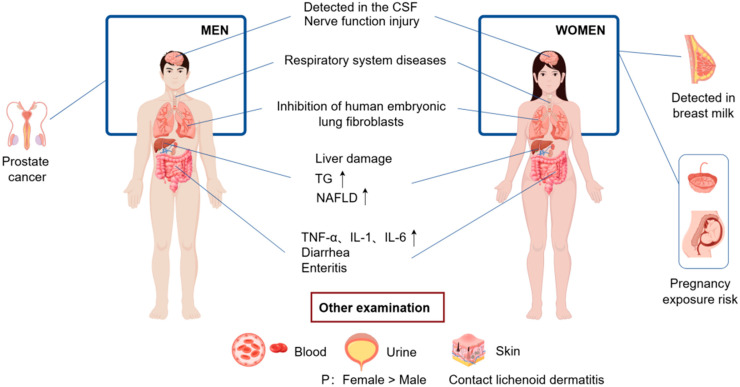
Risk of 6PPD and 6PPDQ to human health. 6PPD and 6PPDQ can be detected in various organs or tissues including blood, urine, cerebrospinal fluid (CSF), milk, and placenta etc. Exposure to high dose of 6PPD and 6PPD increases the level of liver damage biomarker such as triglyceride (TG) and non-alcoholic fatty liver disease (NAFLD), and inflammatory cytokines including tumor necrosis factor-α (TNF-α), interleukin-1 (IL-1), and interleukin-6 (IL-6).

6PPD and 6PPDQ enter the human body through three primary routes—atmospheric inhalation, dietary ingestion, and skin absorption—and subsequently affect the health of multiple systems and organs through a variety of ways (Figure 2).

Firstly, 6PPD and 6PPDQ readily bind to fine particulate matter (PM_2.5_), enabling deep lung deposition and potential translocation across the blood–air barrier into systemic circulation [21]. While PM_2.5_ poses a greater penetration risk, coarse particles (PM_10_) largely trapped in the upper airways still threaten respiratory health under chronic exposure, particularly in vulnerable groups (children, the elderly, and pregnant women) [84]. Additionally, dust-bound 6PPD/6PPDQ can adhere to clothing, later resuspending and entering the body via secondary inhalation or dermal transfer [85].

Secondly, environmental contamination facilitates the uptake of 6PPD and 6PPDQ by crops and aquatic organisms, leading to human exposure through food consumption. These compounds have been detected in fish (aquatic systems) and honey (atmospheric deposition), confirming their bioaccumulative potential [64]. Furthermore, 6PPD migration from rubber food-processing equipment (e.g., milk tubes) into consumables highlights another exposure route [86]. These studies confirmed that 6PPD and 6PPDQ can bioaccumulate and biomagnificate through the food chain.

Third, direct skin contacts with tire wear particles (TWPs) or contaminated dust—common in rubber manufacturing and e-waste recycling—serves as a critical exposure pathway. Workers lacking protective gear often exhibit dermatological effects, including contact dermatitis, eczema, and skin lesions [87,88,89].

Dermal contact represents a significant exposure pathway to 6PPD and 6PPD quinone (6PPDQ), particularly for individuals handling tire wear particles (TWPs) or indoor dust. Occupational exposure studies have demonstrated that these compounds can be absorbed through the skin upon contact with TWPs in outdoor environments or contaminated indoor dust. Workers in rubber manufacturing and e-waste recycling facilities who fail to utilize proper personal protective equipment (PPE), such as gloves and respiratory masks, are particularly vulnerable. Epidemiological evidence documents that such occupational exposure is associated with various dermatological conditions, including pruritus, contact dermatitis, eczema, hyperpigmented lesions, and skin fissures on the hands [87,88,89].

In summary, the detection of 6PPDQ in human urine, blood, and cerebrospinal fluid underscores the inevitability of exposure across all three routes. Given its persistence and bioaccumulation potential, further research is needed to assess long-term health impacts, particularly in high-risk populations.

## 5. Biotoxicity Studies of 6PPD and 6PPDQ

6PPD and 6PPDQ exhibit disruptive effects in organisms through multiple mechanisms, including oxidative stress, inflammatory responses, metabolic disruption, and direct cellular and neurological damage [90,91]. They may cause oxidative stress through the production of reactive oxygen species (ROS) [92], leading to cellular damage, lipid peroxidation, protein oxidation, and DNA damage. In addition, exposure to 6PPD and 6PPDQ may activate inflammatory pathway to release inflammatory cytokines, such as tumor necrosis factor-alpha (TNF-alpha) and interleukins (ILs), thus triggering a local or systemic inflammatory response in humans [60]. Additionally, 6PPDQ is considered genotoxic due to its ability to bind with DNA, which may accelerate DNA adduct formation, DNA strand breaks, and gene mutations, consequently increasing the risk of cancer [27]. Moreover, 6PPD and 6PPDQ are also able to act directly on cell membranes and mitochondria leading to cellular dysfunction [93].

### 5.1. Aquatic Organisms

Up to now, the toxic effects of 6PPD and 6PPDQ are mainly focused on aquatic organisms. According to previous studies, the acute toxicity of 6PPDQ in fish has species specificity. In particular, 6PPDQ is less toxic than silver salmon to zebrafish, Japanese medaka, daphnia, and telopods [94], with silver salmon and zebrafish being the most intensively studied.

#### 5.1.1. Coho Salmon

6PPDQ contamination in urban runoff has been linked to acute mortality in coho salmon in the Pacific Northwest. The 24 h median lethal concentration (LC_50_) for coho salmon is 95 µg/L [47]. Histopathological analyses reveal severe damage to brain tissue and olfactory regions, along with cerebrovascular leakage. Neurochemical alterations include increased dopamine (DA) and gamma-aminobutyric acid (GABA) levels, alongside decreased acetylcholine (ACh) in the brain [2].

#### 5.1.2. Zebrafish

In zebrafish, the 24 h LC_50_ for 6PPDQ is 308 µg/L. Exposure induces intestinal inflammation, evidenced by luminal reddening, likely mediated by oxidative stress [17]. Developmental abnormalities include impaired eye formation, reduced heart rate (indicating cardiotoxicity), and spinal deformities, with a significant increase in spinal curvature (45–90°) at higher concentrations [95,96]. Behavioral studies demonstrate that 6PPDQ impairs locomotor activity, reducing swimming speed and travel distance while increasing angular movement. Additionally, respiratory frequency declines with prolonged exposure [17,54].

These findings underscore the ecological risks posed by 6PPD and 6PPDQ, particularly in urban aquatic environments, necessitating further research on their long-term impacts and mitigation strategies.

### 5.2. Mice

**Figure 3:**
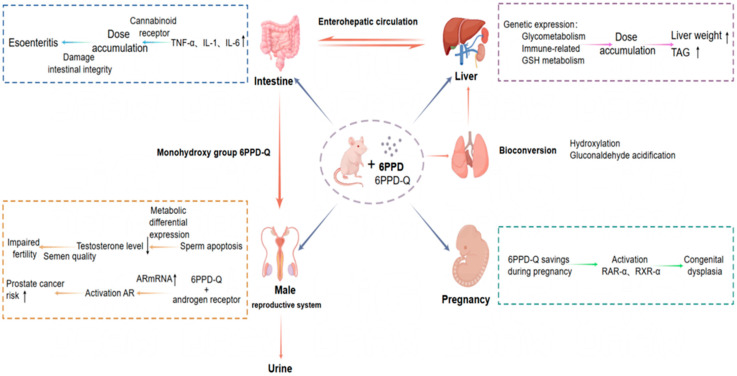
Potential toxicity mechanism of 6PPD and 6PPDQ based on mammals (mouse).

Emerging evidence indicates that 6PPD and 6PPDQ exert multiorgan toxicity in mammals, affecting reproductive, urinary, digestive, and metabolic systems (Figure 3).

Chronic exposure (40 days) to 6PPD and 6PPDQ significantly reduces serum testosterone levels in male mice, impairing spermatogenesis and in vitro fertilization capacity [97]. Additionally, epidemiological and experimental studies suggest a potential association between 6PPD/6PPDQ exposure and prostate cancer development [46]. Zhao et al. reported low urinary excretion of 6PPDQ in exposed mice, indicating significant bioaccumulation [98]). Notably, 6PPDQ exhibits high transplacental transfer efficiency, accumulating in fetal tissues, including the embryonic brain, suggesting potential developmental neurotoxicity.

Oral administration of environmentally relevant doses of 6PPD for 21 days disrupts intestinal barrier integrity in a dose-dependent manner, particularly in the jejunum and ileum [99]. Furthermore, prolonged exposure (6 weeks) induces hepatic steatosis, marked by elevated triglyceride levels and metabolic dysregulation [65]. These findings highlight the systemic toxicity of 6PPD and 6PPDQ, warranting further investigation into their long-term health impacts and regulatory measures.

### 5.3. The Human Body

The toxicological effects of 6PPD and 6PPDQ on organisms have garnered increasing scientific attention. While extensive research has focused on aquatic species, emerging studies have begun to investigate their impacts on human health. Several studies suggest a potential association between 6PPD/6PPDQ exposure and neurological disorders. Notably, Fang et al. reported that brain tissue levels of 6PPD and 6PPDQ in Parkinson’s disease patients were twice those of control subjects, implying a possible link to disease pathogenesis [100]. Additionally, in vitro studies have demonstrated cytotoxic effects of 6PPD on human cells. For instance, Yong et al. observed a dose-dependent inhibition of cell proliferation in human embryonic lung fibroblasts following 24 h 6PPD exposure, as measured by colorimetric assay [101].

Dermal exposure studies indicate that 6PPDQ may contribute to occupational skin conditions. Ancona et al. documented a higher incidence of contact dermatitis-like symptoms among tire manufacturing workers chronically exposed to 6PPDQ [102]. These findings align with earlier reports by Herve-Bazin et al., who noted that rubber antioxidants including 6PPDQ could provoke cutaneous reactions such as erythema and edema [103]. Recent mechanistic research by Chen et al. revealed an inverse correlation between 6PPD/6PPDQ cytotoxicity and their binding affinity to human serum albumin (HSA) [82]. Intriguingly, their study identified opposing effects on HSA enzymatic activity that 6PPD enhanced esterase-like function, whereas 6PPDQ exhibited inhibitory effects.

Despite these findings, significant knowledge gaps remain regarding the toxicological mechanisms of 6PPDQ. Current hypotheses suggest mitochondrial dysfunction as a potential pathway [27,104], though further evidence is required for validation. The limited human toxicological data underscore the need for additional research to assess exposure risks and elucidate molecular mechanisms.

## 6. Health Risk Assessment for Human Exposure to 6PPD and 6PPDQ

In terms of human exposure assessment modeling, some researchers have calculated the daily intake for adults and children via exposure assessment. The formula is as follows.

$${DI}_{der}=\frac{C_{RS}\times CF\times SA\times AF\times ABS\times EF\times ED}{BW\times AT}$$

$${DI}_{ing}=\frac{C_{RS}\times {IR}_{ing}\times CF\times EF\times ED}{BW\times AT}$$

where *DI_der_* and *DI_ing_* represent the daily intake via the dermal and oral routes of dust absorption, respectively.

C_RS_ represents the total concentration of ΣPPDs in dust and the concentration of 6PPDQ (ng/kg).

IR_ing_ represents the ingestion rate of dust (mg/d).

EF stands for exposure frequency (d/a).

ED stands for exposure duration (a).

BW stands for body weight (kg).

AT represents the average exposure time (d).

CF stands for the conversion factor (10^−6^ kg/mg).

SA stands for skin-accessible surface area (cm^2^).

AF stands for the adhesion factor of dust to Fur (mg/cm^2^).

ABS stands for the absorption coefficient (magnitude 1).

The parameters used to assess the daily intake of children and adults are shown in Table 7 [96,105].

Several studies have quantified the exposure levels of 6PPD and 6PPD quinone (6PPDQ) in children from e-waste dismantling regions (e.g., Guiyu) compared to reference areas (e.g., Haojiang). Exposure levels were assessed by estimating the daily intake (EDI) of polybrominated pollutants. The estimated daily intake (EDI) of children exposed to 6PPDQ, PBDEs, PCBs and heavy metals present in kindergarten dust was calculated via the following formula [106]:

$$EDI={(C}_{i}\times {TF}_{i}\times IR)/BW$$

where C_i_ is the concentration (ng/g) of 6PPDQ, PBBs, PCBs, or heavy metals (types) in kindergarten dust;

TF_i_ stands for children’s exposure time in kindergarten, where children spend an average of approximately 8 h per day;

IR refers to the average daily dust intake, which is 0.05 g/day for children. BW stands for the body weight (kg) of the child.

Bayesian kernel machine regression revealed that the EDIs of these pollutants correlated with altered gut microbiota composition, suggesting a potential mechanistic link between exposure and metabolic disruption.

The daily urinary excreta of the children were calculated via a formula [9].

de=C×V/W

where C (ng/mL) is the concentration of 6PPD and 6PPDQ in the child’s urine; V (mL/day) is the amount of urine excreted by the child per day; and W (kg) is the child’s weight.

The results revealed that children in the e-waste dismantling area had 10-fold higher urinary levels of both compounds than those in the reference area [9]. Notably, 6PPDQ concentrations exceeded 6PPD by an order of magnitude, aligning with its greater bioaccumulative potential and toxicity. Additionally, older children exhibited higher exposure levels, likely due to increased outdoor activity time [98]. This may be related to the fact that older children spend more time outdoors.

Further research evaluated 6PPD/6PPDQ exposure risks among 925 kindergarten children in an e-waste dismantling area and a reference area by measuring airborne and dust concentrations in kindergartens, households, and roadside environments. Daily intake (DI) was estimated via ingestion and inhalation pathways using the following formulas [83].

$${DI}_{ingestion}=\frac{C\times IngR\times CF}{Bw}$$

$${DI}_{inhalation}=\frac{C\times InhR}{PEF\times BW}$$

$${DI}_{both}={DI}_{ingestion}+{DI}_{inhalation}$$

Here, C (ng/kg) is the concentration of 6PPD and 6PPDQ from home or kindergarten dust (ng/kg); IngR is the ingestion rate (100 mg/day); InhR is the inhalation rate (13.3 m^3^/day); CF is the conversion factor (10^–6^ kg/mg); BW is the body weight (kg); and PEF is the dust–air particle emission factor (m^3^/kg).

Children in the e-waste dismantling area faced significantly higher exposure levels than those in the reference area. Epidemiological data from 2019 indicated that Guiyu children had lower BMI, higher rates of diarrhea and influenza, and altered gut microbiota compared to controls. These findings suggest that chronic 6PPD/6PPDQ exposure may compromise immune function and growth development [83].

Human exposure to 6PPD and 6PPDQ occurs through diet, respiration, and dermal contact, with e-waste recycling zones posing elevated risks. The heightened toxicity of 6PPDQ, combined with evidence of metabolic and immunological disruptions in children, underscores the urgent need for interventions to mitigate health hazards in vulnerable populations [9,83].

## 7. Prevention and Intervention

To minimize the adverse health effects of 6PPD and its highly toxic quinone derivative (6PPDQ), interventions should target source control, exposure reduction, and safer alternatives.

6PPD partially decomposes during vulcanization, and subsequently generates toxic aromatic amines, which should promote research to focus on less hazardous substitutes or develop safer antioxidant alternatives [107]. RU 997, proposed by Krüger et al., demonstrates potential as a viable 6PPD alternative [108]. Huntink et al. prepared a new antioxidant by graft copolymerization of N (4-phenylenediamine) maleimide into natural rubber [109]. In addition, 6PPD-related derivatives, carboxylates, etc., can be substituted as new antioxidants. Compared with conventional antioxidants, these improved rubbers have superior properties [22], are more efficient against ozone, and some are even able to reduce the skin-sensitizing potential [109], reduce the degree of cytotoxicity to skin cells, and minimize skin penetration. The use of safer antioxidant alternatives can reduce their exposure to unprotected populations.

In addition, wastewater treatment can be strengthened to prevent high levels of 6PPD and 6PPDQ drinking water from being ingested by individuals; it is also crucial to take proper personal protective measures, such as wearing appropriate protective gear for workers in e-waste dismantling factories or workers in rubber factories during their daily work, as well as the use of air purifiers in indoor environments. Moreover, regular health check-ups and biomonitoring will enable possible health problems to be detected and addressed in a timely manner. Through these measures, the potential toxic effects of 6PPD and 6PPDQ on humans can be effectively minimized, and public health can be protected. In areas with high concentrations of 6PPD and 6PPDQ, such as e-waste dismantling zones, scientific management to promote traditional e-waste recycling to scientific emerging recycling zones is a very effective way to reduce the concentrations of 6PPD and 6PPDQ.

## 8. Conclusions

This study reviews the environmental distribution, the human exposure pathways, the toxic effects, and health risk assessment models of 6PPD and 6PPDQ. 6PPD and 6PPDQ accumulate in the human body through various pathways, ultimately posing a threat to human health. Thus far, there is relatively less research on the direct impact on human health. Therefore, future research needs to focus more on the distribution of 6PPD and 6PPDQ in the human body. Meanwhile, the toxicological mechanisms of 6PPD and 6PPDQ on the human body should be strengthened. In addition, it is extremely important to develop more scientific and rigorous human exposure assessment models to provide early warnings to susceptible populations. Furthermore, it is necessary to develop effective degradation technologies, such as the advanced oxidation system of sunlight-activated periodate, or monitor the 6PPD and 6PPDQ exposure levels in the human body. However, several limitations remain in this study such as lack of standardized methods and scarcity of human exposure data. With the increase in the understanding of the environmental behaviors and health impacts of 6PPD and 6PPDQ, the emergence of more effective preventive measures and intervention strategies can be expected to reduce the impact on human health in the future. Treatment and mitigation strategies for 6PPD and 6PPDQ span their entire lifecycle. This includes source control (e.g., formulating sustainable rubber antioxidants), pathway interception (e.g., optimizing stormwater management practices), and end-of-pipe treatment (e.g., employing advanced oxidation or adsorption technologies to degrade or remove 6PPD/Q from aqueous environments).

## Figures and Tables

**Table 2:** Half lethal concentration LC50 of 6PPD and 6PPDQ across different species.

Species		Endpoint	Pollutants	LC50 (μg/L)	Test Duration	References
Salvelinus leucomaenis pluvius		Death	6PPDQ	0.51	24 h	[41]
Oncorhynchus kisutch	Infancy	Death	6PPDQ	0.95	24 h	[42]
	Adult	Death	6PPDQ	0.41	24 h	
Oncorhynchus kisutch		Death	6PPDQ	0.804	24 h	[43]
Oncorhynchus masou		Death	6PPDQ	<3.8	24 h	[41]
Brachymystax lenok		Death	6PPDQ	<3.8	24 h	[41]
Salvelinus leucomaenis		Death	6PPDQ	5.1	24 h	[44]
Salvelinus fontinalis		Death	6PPDQ	5.9	24 h	[45]
Oncorhynchus mykiss		Death	6PPDQ	19.6	24 h	[45]
		Death	6PPDQ	9.0	24 h	[46]
Oncorhynchus masou		Death	6PPDQ	>100	24 h	[44]
Salvelinus alpinus		Death	6PPDQ	>127	24 h	[45]
Psephurus gladius		Death	6PPDQ	>127	24 h	[45]
Oryzias latipes		Death	6PPDQ	>340	96 h	[47]
Danio rerio larva		Death	6PPD	1384.93	24 h	[17]
				442.62	96 h	
			6PPDQ	308.67	24 h	
				132.92	96 h	
Danio rerio		Death	6PPDQ	>540	96 h	[47]
		Death	6PPDQ	3086.7	96 h	[17]
Oncorhynchus tshawytscha		Death	6PPDQ	>673.06	24 h	[42]
		Death	6PPDQ	>800	24 h	[43]
Collembola		Death	6PPDQ	16.31 (μg/kg) in soil	28 d	[48]
Caenorhabditis elegans		Death	6PPDQ	>100	4.5 d	[49]

**Table 3:** The concentrations of 6PPD and 6PPDQ in water in the world.

Environmental Media	6PPDQ	6PPD	Countries and References
Surface water			
City surface water	96–112 ng/L	/	Canada [53]
Zhujiang River	1.51 (0.26–11.3) ng/L	0.48 (0.31–1.07) ng/L	China [54]
Dongjiang River	0.91 (0.29–8.12) ng/L	0.36 (0.27–1.29) ng/L	
Liuxi River	0.18 (/–0.75) ng/L	/	
Brisbane River	17.5 (0.38–88) ng/L	/	Australia [55]
Jiaojiang	6.1 (<LOD–21) ng/L	10 (4.0–72) ng/L	China [16]
Stormwater			
A city with heavy traffic	1.12 (0.21–2.43) µg/L	0.32 (0.21–2.71) µg/L	China [27]
Tunnel wash runoff	49.5–143 ng/L	/	Norway [56]
Artificial turf runoff	159 ng/L	/	
City stormwater	48–5580 ng/L	/	Canada [53]
Roadway runoff	576 (38.5–1562) ng/L	3.05 (0.41–7.52) ng/L	China [54]
Courtyard runoff	51.6 (6.03–875) ng/L	0.89 (0.19–1.10) ng/L	
Farmland runoff	0.73 (0.53–5.58) ng/L	/	
Groundwater			
Guangzhou	0.11 (/–0.70) ng/L	/	China [54]
Waste water			
Influent (raw)	53 (1.9–470) ng/L	12 (1.1–59) ng/L	China [57]
Effluent (treated)	3.4 (1.1–37) ng/L	0.30 (<LOQ–15) ng/L	
Influent (raw)	64.8 ± 5.3–145.7 ± 46.7 ng	/	Canada [51]
Effluent (treated)	<LOD–446.5 ± 37.7 ng	/	
Influent (raw)	777 (592–1100) ng/L	/	Germany [58]
Effluent (treated)	50 (41–66) ng/L	/	
Malaysia WWTP influent (raw)	/	/	Malaysia and Sri Lanka [59]
Malaysia WWTP effluent (treated)	/(/–0.11) ng/L	/	
Sri Lanka WWTP influent (raw)	/(/–0.37) ng/L	/	
Sri Lanka WWTP effluent (treated)	/(/–0.37) ng/L	/	
WWTP influent (raw)	14.2 ± 0.80 to 69.8 ± 2.40 ng/L	/	China [60]
WWTP effluent (treated)	/–2.09 ± 0.16 ng/L	/	
Snowmelt period WWTP influent (raw)	0.105 ± 0.037 µg/L	4.4 µg/L	Germany [61]
Snowmelt period WWTP Effluent (treated)	/	2.4 µg/L	
Rainfall period WWTP influent (raw)	0.052 ± 0.022	14.3	
Rainfall period WWTP effluent (treated)	/	11.2	
Dry weather WWTP influent (raw)	/	0.9	
Dry weather WWTP effluent (treated)	/	0.3	
Drinking water			
Singapore	/	<10 ng/L	Singapore [62]
Snowmelt			
City	2019: 367 (74–756) ng/L	/	Canada [63]
City	2020: 81 (15–172) ng/L	/	
Snow			
Roadside	259 (110–428) ng/L	329 (/–784) ng/L	Germany [58]

**Table 4:** The concentrations of 6PPD and 6PPDQ in aquatic organisms and food in the world.

	Species	6PPDQ	6PPD	Countries/Regions and References
Aquatic organism	Snakehead	/	0.669 μg/kg	China [64]
	Weever	/	0.481 μg/kg	
	Spanish mackerel	<LOQ	/	
	Zebrafish	/	351 ng/g	China [65]
	Rainbow trout	BCFs of 6PPDQ were calculated as 2.9, 19, 25, and 17.2	/	Canada [66]
		293 L/kg at the water concentrations of 0.8, 3, 12, and 25 µg/L		
	Rotundipterus	/	1206 pg/g	Norway [67]
	Zebra fish	Max of 225 at 48 h	Max of 3000 at 48 h	Germany [68]
Food	Lettuce-1 mg/L6PPDQ	2.19 µg/g	0.78 µg/g	Austria [69]
	Lettuce-TWP	0.02 µg/g	0.4 µg/g	
	Honey	/	/	China [64]

**Table 5:** The concentrations of 6PPD and 6PPDQ in soil and sediment in the world.

Environmental Media	Type	6PPDQ	6PPD	Countries/Regions and References
Soil	Roadside	234 (9.50–936) ng/g	309 (31.4–831) ng/g	China [Hong Kong, New Territories and Kowloon] [27]
Sediment	Fluvial sediment	9.03 (1.87–18.2) ng/g	14.4 (0.585–468) ng/g	China [Pearl River Delta, Pearl River Estuary, South China Sea] [73]
	Estuarine sediment	2.00 (<MDL–4.88) ng/g	3.9 (1.49–5.71) ng/g	
	Coast sediment	1.27 (0.431–2.98) ng/g	1.82 (1.07–11.1) ng/g	
	Abyssal sediment	2.71 (<MDL–3.02) ng/g	2.66 (<MDL–2.69) ng/g	

**Table 6:** The distributions of 6PPD and 6PPD quinone in different environments.

Environmental Media	Main Content	References
Water Environment	The occurrence and partitioning of p-phenylenediamine antioxidants and their quinone derivatives in water and sediment.	[16]
	The toxicological effects of 6PPD and 6PPD quinone on zebrafish larvae.	[17]
	6PPD quinone and its emergence as a new threat to aquaculture and fisheries.	[41]
	The detection of specific tire wear compounds in urban receiving waters.	[51]
	6PPD and its metabolite 6PPDQ cause different developmental toxicities and phenotypes in embryonic zebrafish.	[60]
	Conducting toxicity and mutagenicity studies of 6PPD quinone in a marine invertebrate species as well as in bacteria.	[75]
	Comparing the toxic effects of 6PPD and 6PPD quinone, which are compounds derived from tire wear particles, on Chlorella vulgaris.	[76]
	The exposure of 6PPD and 6PPDQ in rivers in the United States.	[77]
	The impacts of environmental concentrations of 6PPD and its quinone metabolite on the growth and reproduction of freshwater cladoceran.	[78]
Soil Environment	The enhanced formation of 6PPDQ during the aging of tire wear particles in anaerobic flooded soils and explores the roles of iron reduction and environmentally persistent free radicals in this process.	[48]
	The nationwide presence and prioritization of tire additives and their transformation products in lake sediments across China.	[50]
	The temporal and spatial accumulation of 6PPDQ in green belt soil and its effects on soil microbial community were studied.	[72]
	The responses of soil and collembolan (Folsomia candida) gut microbiomes to 6PPDQ pollution.	[79]
Atmospheric Environment	The relevant research content on comprehensively characterizing the tire and road wear particles in the road dust of the highway tunnel by using the size and density fractionation method.	[6]
	The role of p-phenylenediamine-derived quinones as new contributors to the oxidative potential of fine particulate matter, discussing their presence, distribution, and correlations in PM_2_.	[10]
	The formation of transformation products during the heterogeneous ozonation of the tire rubber antioxidant 6PPD.	[11]
	Six PPD-derived quinone compounds and eight PPD antioxidants simultaneously assessed in PM_2.5_.	[20]
	The association between particulate matter pollution (PM_2.5_) in the atmosphere of 652 cities around the world and daily mortality and found that there were regional differences.	[21]
	The development of new hair dyes as alternatives to the hazardous para-phenylenediamine.	[22]
	The widespread occurrence and distribution characteristics of 6PPDQ in size-fractioned atmospheric particles and dust from different indoor environments.	[23]
	The occurrence of substituted p-phenylenediamine antioxidants in dust.	[31]
	The particle size distribution of rubber tire-related chemicals in road and indoor parking lot dust.	[35]
	Association between 6PPDQ exposure and body mass index (BMI), influenza, and diarrhea in children.	[74]
Comprehensive	The environmental profiles, hazard identification, and toxicological hallmarks of the emerging tire rubber-related contaminants 6PPD and 6PPD quinone.	[1]
	The sources of p-phenylenediamine antioxidants (PPDs) and their derived quinone compounds (PPDQs), their distribution in environmental media and the human body, human exposure levels, and health risks.	[9]
	The transformation, environmental distribution, bioavailability, and toxicity of the tire-rubber related pollutant 6PPD quinone.	[19]
	The article presents new evidence regarding rubber-derived quinones in water, air, and soil.	[27]
	E-Waste recycling emits large quantities of emerging aromatic amines and organophosphites, which are a poorly recognized source for two classes of synthetic antioxidants.	[30]
	N-(1,3-Dimethylbutyl)-N′-phenyl-p-Phenylenediamine (6PPD) and its derivative 6PPD quinone in the environmental context.	[80]
	The environmental fate of tire-rubber related pollutants 6PPD and 6PPDQ.	[81]
	The analysis methods, environmental occurrence, fate in the environment, and potential toxicity of tire wear compounds 6PPD and 6PPD quinone.	[82]
E-Waste	Concentration, source, and health effects assessment in urine, involving multiple environmental mediators in e-waste dismantling areas.	[9]
	Temporal and spatial variations in 6PPD and 6PPDQ in dust and soil of e-waste recycling area were studied, involving the environmental behavior and potential risks of 6PPDQ in various environmental media.	[74]
	The changes in gut microbiota and its metabolomics in children who are exposed to 6PPDQ, PBDE, PCB, and metal(loid).	[83]

**Table 7:** Parameters used to estimate human exposure to PPDs and 6PPDQ.

Parameter	Unit	Children	Adult
Ingestion rate (IR_ing_)	mg/d	200	100
Exposure frequency (EF)	d/a	365	365
Exposure duration (ED)	a	6	24
Body weight (BW)	kg	16.58	58.55
Average time during exposure (AT)	d	365 × 70	365 × 70
Conversion factor (CF)	kg/mg	10^−6^	10^−6^
Skin surface area available for contact (SA)	cm^2^	1150	2145
Soil-to-skin adherence factor (AF)	mg/cm^2^	0.2	0.07
Adsorption factor (ABS)	%	13	13

## Data Availability

The data presented in this study are available on request from the corresponding author.
